# Prevalence of constipation in adults with obesity class II and III and associated factors

**DOI:** 10.1186/s12876-021-01806-5

**Published:** 2021-05-12

**Authors:** Erika Aparecida Silveira, Annelisa Silva e Alves de Carvalho Santos, Jessivane Nascimento Ribeiro, Matias Noll, Ana Paula dos Santos Rodrigues, Cesar de Oliveira

**Affiliations:** 1 Faculty of Medicine, Postgraduate Program in Health Sciences, Federal University of Goias, Goiania, Brazil; 2 Affiliate Academic, Department of Epidemiology and Public Health, University College London, London, UK; 3 Federal Institute Goiano, Ceres, Brazil; 4 Department of Epidemiology and Public Health, University College London, London, UK

**Keywords:** Constipation, Dietary habits, Polypharmacy, Functional gastrointestinal disorders, Morbid obesity

## Abstract

**Background:**

Constipation and obesity have common risk factors. However, little is known about the occurrence of constipation in individuals with severe obesity and the associated factors.

**Objective:**

To evaluate the prevalence of intestinal constipation and its associated factors in adults with obesity class II and III.

**Method:**

This study analyzed baseline data from a randomized clinical trial with adults aged 18–64 with a Body Mass Index (BMI) ≥ 35 kg/m^2^, living in the metropolitan region of Goiânia, Brazil. Data were collected using a questionnaire containing sociodemographic, lifestyle, level of obesity, presence of comorbidities, water intake and food consumption variables. The outcome variable was constipation assessed by the Rome III criteria and the Bristol Stool Form Scale. Multiple Poisson regression analysis was used to assess the association between explanatory variables and the outcome.

**Results:**

Among the 150 participants, the prevalence of constipation was 24.67% (95% CI: 17.69–31.64). After multiple regression analyses constipation was associated with polypharmacy (adjusted PR: 2.99, 95% CI: 1.18–7.57, *p* = 0.021), younger age group i.e. 18–29 years (adjusted PR: 3.12, 95% CI: 1.21–8.06, *p* = 0.019) and former smoking (adjusted PR: 3.24, 95% CI: 1.28–9.14, *p* = 0.014). There was no statistically significant association between constipation and daily consumption of fiber-rich foods, however, the non-consumption of whole grains was borderline significant (adjusted PR: 2.92, 95% CI: 1.00 to 8.49, *p* = 0.050).

**Conclusion:**

A high prevalence of constipation was found in adults with obesity class II and III. Constipation was significantly associated with the simultaneous use of five or more medications, younger age group and being a former smoker.

## Background

Constipation is a chronic problem that affects many individuals worldwide [1], especially older adults [2]. Intestinal constipation (IC) is a disorder of the gastrointestinal tract defined as an unsatisfactory bowel movement, characterized by difficulty in defecating, low frequency of bowel movements, occurrence of painful bowel movements, hard stools or feeling of incomplete bowel movement [3, 4]. Globally, it is estimated that the prevalence of constipation in adults is approximately 16% [3] and millions of dollars are spent annually on the use of laxatives. The prevalence in adults in Australia assessed by the criteria of Rome III was 24% [5]. In Brazil, previous studies have demonstrated that the prevalence of constipation ranged between 14 and 26% [6–8]. Some studies identified risk factors for the occurrence of constipation in the general population: female gender, advanced age, low socioeconomic status, physical inactivity, diabetes, medication and dietary factors, such as low fiber consumption, low fluid intake and high consumption of fast foods [1, 9]. Moreover, constipation may also be associated with lower quality of live and mental issues [10, 11]. Additionally, constipation in women was associated with hormonal disorders and obesity [2, 12–16]. However, little is known about the factors associated with constipation in individuals with class II and III obesity.

Obesity is a multifactorial chronic disease characterized by excessive accumulation of body fat and with high prevalence in adults of both sexes [17–19]. A body mass index (BMI) greater than or equal to 35 kg/m^2^, that is, classes II and III, increases the risk of developing or worsening other chronic diseases and mortality, being a serious public health problem [20, 21]. Obesity classes II and III has shown a progressive and greater increase than the other obesity levels globally. Research on BMI trends in more than 200 countries between 1974 and 2014 demonstrated a global prevalence of obesity classes II and III of 2.3% among men and 5.0% among women [20]. In Brazil, the prevalence of class III obesity increased by 36.4% between 2006 and 2013, reaching 1.5% of the population [22].

Obesity is a risk factor for diabetes, hypertension, cardiovascular disease and more than thirteen types of cancer [21, 23, 24]. Constipation is also a risk factor for cancer [25], especially gastrointestinal cancer, including esophagus, stomach, small intestine, liver and pancreas [9, 26, 27]. Obesity is associated to constipation, but it is not recognized as a causal factor of constipation. A recent study has showed that obese individuals have several other risk factors for constipation, such as physical inactivity, low quality of their diet with low consumption of fibers and vegetables in general [26]. Constipation and obesity have common risk factors. However, little is known about the occurrence of constipation in individuals with severe obesity and the associated factors [28–31]. A recent review of studies on constipation did not report the prevalence and associated factors in individuals with obesity [3, 4, 16, 32]. The few studies available address strategies for the treatment of constipation in obese individuals [31, 33]. Therefore, it is important to establish the magnitude of the occurrence of the problem and the associated factors to design treatment strategies, especially considering that nutritional interventions leading to a healthy diet, improved eating habits and lifestyle can treat both constipation and obesity. Considering the lack of evidence on constipation in individuals with obesity class II and III and the relevance of this problem, the aim of this research was to evaluate the prevalence of constipation and its associated factors in adults with class II and III obesity and to describe their intestinal habits profile.

## Methods

### Study design and ethical aspects

This study analyzed the baseline data from the randomized clinical trial with severely obese individuals entitled “Effect of nutritional intervention and olive oil on severe obesity—DieTBra Trial”. Details of the study design, subject recruitment and randomization were previously described [34–39]. The main project was approved by the Research Ethics Committee of Clinical Hospital, Federal University of Goiás (protocol number 747.792). All individuals who met the inclusion criteria and agreed to participate in the research signed an informed consent form.

### Inclusion and exclusion criteria

This study included adults with obesity class II and III (Body Mass Index (BMI) ≥ 35 kg/m^2^) attending the primary care network of the Brazilian National Health System (SUS). They were referred by the Municipal Health Department to the Outpatient Clinic of Nutrition in Severe Obesity (CNSO) at the Clinical Hospital of the Federal University of Goias. At the time of data collection, CNSO was the only reference clinic in the treatment of severe obesity in the metropolitan region of Goiânia. Inclusion criteria were age between 18 and 64 years, both sexes and residence in the metropolitan region of Goiânia. The exclusion criteria were individuals who have undergone bariatric surgery, a reduction of more than 8% of body weight in the last three months, being on medication for weight loss, pregnant or lactating women and people with special needs.

### Data collection and study variables

The data collection was carried out by trained nutritionists. The questionnaire contained sociodemographic variables (sex, age, schooling years, social class, marital status and skin color); lifestyle (physical activity, smoking and alcohol consumption i.e. grams of ethanol ingested); level of obesity [21] (class II: BMI between 35.0 and 39.9 kg/m^2^; class III: BMI between 40.0 and 49.99 kg/m^2^; and super obesity [40]: BMI > 50.0 kg/m^2^); self-reported morbidities (diabetes, hypertension, dyslipidemia, biliary lithiasis, depression, anxiety, hiatus gastritis/hernia and gastroesophageal reflux); daily water intake, frequency of consumption of raw salad, cooked/braised vegetables, fresh fruits, and whole grains.

The level of physical activity was assessed by the Global Physical Activity Questionnaire (GPAQ) that was developed by the World Health Organization, with a cut-off point for classifying individuals as active when they reached more than 150 min in moderate activities or more than 75 min of intense activity in a typical week [41]. Alcohol consumption was assessed using a questionnaire adapted from the Gender, Alcohol and Culture: an International Study (GENACIS study). For the conversion of habitual alcohol consumption to grams of ethanol, 13 g of ethanol were standardized per drink or dose of alcoholic beverage [42].

The polypharmacy variable was defined as the use of five or more medications [43]. The multimorbidity variable was constructed considering 18 self-reported doctor-diagnosed health conditions [44] (diabetes; hypertension; dyslipidemia; osteoporosis; stroke; cardiovascular disease—atherosclerosis, heart failure, infarction; respiratory disease—asthma, bronchitis; sleep apnea; arthritis/arthrosis; thyroid dysfunction—hyper/hypothyroidism; liver disease—liver steatosis, cirrhosis; gastroesophageal reflux; urinary incontinence; cancer; infertility; varicose veins). The presence of multimorbidity was defined as two or more self-reported conditions [45]. Anxiety and depression were assessed using the validated Hospital Anxiety and Depression Scale (HAD) [46].

Data on food intake were collected using an adapted version of the Food Frequency Questionnaire (FFQ) developed by Furlan-Viebig and Pastor-Valero [47] for an adult population, assessing habitual consumption in the last year, with frequency of weekly consumption (1×/week, 2–3×/week, 4–6×/week, daily), monthly (1×/month, 2×/month, 3×/month) or rare consumption (< 1× /month). For analysis purposes, the frequency of consumption of the food/food group of interest was categorized into daily consumption (yes or no). Daily water consumption was computed in number of glasses per day, later converted to liters (L) and categorized as < 2 L, 2 L and > 2 L/day.

### Intestinal constipation

In the present study, IC was assessed according to the Rome III criteria, defined by the presence of at least two of the following clinical manifestations in the three months prior to the interview: evacuation effort in > 25% of evacuations; feeling of incomplete bowel movement in > 25% of bowel movements; less than three bowel movements a week; sensation of exit obstruction in > 25% of bowel movements; and manual evacuation-facilitating maneuvers in > 25% of evacuations [5, 48, 49].

The frequency of bowel movements per week, hard stools and the need for excessive effort to evacuate were also assessed. Weekly evacuation frequency was defined as follows: 5 or more as normal frequencies, 3–4 moderate frequencies and fewer than 3 irregular/abnormal frequencies [50]. Stool consistency was assessed using the original version of the Bristol Stool Form Scale [51], composed of combined and standardized graphic and descriptive methods representing seven types of stools with different shapes and consistencies.

### Statistical analysis

The results were expressed in absolute and relative frequencies. Pearson's χ^2^ test or Fisher's Exact test with an alpha of 5% were used to analyze the association between variables. Prevalence and prevalence ratios were calculated with their 95% confidence intervals. Multiple Poisson regression analysis with robust variance was performed with those variables showing a *p* < 0.20 in the bivariate analysis, namely: polypharmacy, smoking, age, daily consumption of whole grains, presence of dyslipidemia and ingested grams of ethanol. The database was structured in the EPI DATA® version 3.1 program, with double entry of data for subsequent analysis of consistency and quality assurance of information. All analyzes were performed using the STATA® version 16.0 program (Stata Corp, College Station, TX).

## Results

Among the 150 adults with class II and III obesity who participated in the present study, the prevalence of IC was 24.7% (95% CI: 17.69–31.64). Ten individuals (6.7%) had fewer than 3 weekly evacuations and 14% reported 3–4 times, both within the highest risk of constipation (*p*-value = 0.000). These who reported 3–4 bowel movements per week, 61.9% had constipation. Hardened stools or balls in all bowel movements affected 20.7% of the participants and the need for excessive effort to evacuate was 34.0%. The most frequent type of feces according to the Bristol Scale was type 4 with 49.3%. All variables in Table 1 were associated to IC (Table 1).

**Table 1:** Prevalence of intestinal constipation and variables related to bowel movements in individuals with obesity class II and III (n = 150)

Variables	Total	Constipation	*p*-value**
	n (%)	n (%)	
Intestinal constipation			
No	113 (75.33)		
Yes	37 (24.67)		
Weekly evacuations			0.000
5–7 times	119 (79.33)	18 (15.13)	
< 1–4 times*	31 (20.67)	19 (61.29)	
Frequency of hardened stools or stools in balls (n = 58)			0.000
Always	13 (22.41)	10 (76.92)	
At least once a week	27 (46.55)	22 (81.48)	
Every two weeks/monthly/almost never	18 (31.03)	3 (16.67)	
Need of excessive effort to evacuate			0.000
No	99 (66.00)	6 (6.06)	
Yes	51 (34.00)	31 (60.78)	
Bristol stool form scale			0.000***
Type 1	3 (2.00)	3 (100.00)	
Type 2	7 (4.67)	5 (71.43)	
Type 3	22 (14.67)	15 (68.18)	
Type 4	74 (49.33)	11 (14.86)	
Type 5	12 (8.00)	2 (16.67)	
Type 6	23 (15.33)	1 (4.35)	
Type 7	9 (6.00)	0 (0.00)	

*Include one person with less than one time per week

**Pearson’s χ^2^. ***Fisher’s exact test

Higher prevalence of IC was found in females (26.6%), in the younger age group (between 18 and 29 years of age, 47.4%) and among those participants with lower level of education (27.4%). There was a significant association of IC with the younger age group (PR: 2.70, 95% CI: 1.29–5.65) (Table 2).

**Table 2:** Prevalence of intestinal constipation and its association with sociodemographic variables in adults with obesity classes II and III (n = 150)

Variables	Frequency	Prevalence of constipation	PR (CI95%)	*p*-value
	n (%)	n (%)		
Sex				0.285**
Male	22 (14.67)	3 (13.64)	1	
Female	128 (85.33)	34 (26.56)	1.95 (0.65–5.82)	
Age group				0.044*
18–29 years	19 (12.67)	9 (47.37)	2.70 (1.29–5.65)	
30–39 years	57 (38.00)	10 (17.54)	1	
40–49 years	53 (35.33)	11 (20.75)	1.18 (0.55–2.56)	
≥ 50 years	21 (14.00)	7 (33.33)	1.90 (0.83–4.35)	
Years of education				0.809**
1–9	62 (41.33)	17 (27.42)	1.37 (0.52–3.61)	
10–12	68 (45.33)	16 (23.53)	1.18 (0.44–3.13)	
13–15	20 (13.33)	4 (20.00)	1	
Social class				0.956*
A/B	34 (22.67)	9 (26.47)	1.11 (0.57–2.16)	
C	92 (61.33)	22 (23.91)	1	
D/E	24 (16.00)	6 (25.00)	1.04 (0.48–2.29)	
Marital status				0.654**
Single	39 (26.00)	8 (20.51)	1.09 (0.33–3.62)	
Married	95 (63.33)	26 (27.37)	1.46 (0.50–4.28)	
Widowed/divorced	16 (10.67)	3 (18.75)	1	
Skin color				0.346*
White	46 (30.67)	8 (17.39)	1	
Brown	83 (55.33)	24 (28.92)	1.66 (0.81–3.40)	
Black	21 (14.00)	5 (23.81)	1.37 (0.51–3.70)	

^*^Pearson's χ^2^ test. ** Fisher's exact test. PR: prevalence ratio. 95%CI: 95% confidence interval. Bold: significative result

Among the lifestyle variables, being a former smoker (PR: 2.10, 95% CI: 1.18–3.76) and smokers (PR: 2.49, 95% CI: 1.07–5.80) were significantly associated with IC compared to non-smokers. The level of obesity and the presence of morbidities were not associated with IC. An association was also observed between IC and polypharmacy (PR: 2.18, 95% CI: 1.26–3.77) (Table 3).

**Table 3:** Prevalence of intestinal constipation and its association with lifestyle variables and presence of morbidities in adults with obesity class II and III (n = 150)

Variables	Frequency	Prevalence of constipation	PR (CI95%)	*p*-value
	n (%)	n (%)		
Physical activity				0.595*
Sedentary	28 (18.67)	8 (28.57)	1.20 (0.62–2.34)	
Active	122 (81.33)	29 (23.77)	1	
Smoking status				0.015**
No smoker	101 (67.33)	18 (17.82)	1	
Ex-smoker	40 (26.67)	15 (37.50)	2.10 (1.18–3.76)	
Smoker	9 (6.00)	4 (44.44)	2.49 (1.07–5.80)	
Alcohol consumption				0.916
No	25 (17.24)	6 (24.00)	1	
Yes	120 (82.76)	30 (25.00)	1.04 (0.48–2.24)	
Ingested grams of ethanol (n = 80)				0.172*
3–14.99 g	20 (25.00)	7 (35.00)	1.75 (0.80–3.85)	
≥ 15 g	60 (75.00)	12 (20.00)	1	
Obesity level				0.580*
Class II (35.0–39.99 kg/m^2^)	25 (16.67)	7 (28.00)	1.31 (0.62–2.77)	
Class III (40.0–49.99 kg/m^2^)	84 (56.00)	18 (21.43)	1	
Super obesity (> 50 km/m^2^)	41 (27.33)	12 (29.27)	1.37 (0.73–2.56)	
Diabetes				0.249*
No	123 (82.00)	28 (22.76)	1	
Yes	27 (18.00)	9 (33.33)	1.46 (0.78–2.74)	
Arterial hypertension				0.211*
No	66 (44.00)	13 (19.70)	1	
Yes	84 (56.00)	24 (28.57)	1.45 (0.80–2.63)	
Dyslipidemias				0.093*
No	80 (55.17)	15 (18.75)	1	
Yes	65 (44.83)	20 (30.77)	1.64 (0.91–2.95)	
Biliary lithiasis				0.870*
No	122 (82.43)	30 (24.59)	1.07 (0.49–2.30)	
Yes	26 (17.57)	6 (23.08)	1	
Gastritis/Hiatus Hernia				0.714*
No	93 (62.00)	22 (23.66)	1	
Yes	57 (38.00)	15 (26.32)	1.11 (0.63–1.97)	
Gastroesophageal reflux				0.672*
No	125 (83.33)	30 (24.00)	1	
Yes	25 (16.67)	7 (28.00)	1.17 (0.58–2.36)	
Depression				0.313*
No	55 (36.67)	11 (20.00)	1	
Yes	95 (63.33)	26 (27.37)	1.05 (0.58–1.91)	
Anxiety				0.427*
No	26 (17.33)	8 (30.77)	1.32 (0.68–2.55)	
Yes	124 (82.67)	29 (23.39)	1	
Multimorbidity				0.335**
No	14 (9.33)	5 (35.71)	1.52 (0.70–3.27)	
Yes	136 (90.67)	32 (23.53)	1	
Use of laxatives				
No	148 (98.67)	37 (25.00)		
Yes	2 (1.33)	0 (0.00)		
Polypharmacy				0.005*
No	101 (67.33)	18 (17.82)	1	
Yes	49 (32.67)	19 (38.78)	2.18 (1.26–3.77)	

*Pearson's χ^2^ test. **Fisher's exact test. PR: prevalence ratio. 95%CI: 95% confidence interval

Bold: significative result

Regarding the consumption of water and dietary sources of fibers, although 28.6% of the participants consumed less than two liters of water per day and the low frequency of individuals with daily consumption of raw salad (20.3%), cooked/braised vegetables (19.5%), fresh fruits (22.5%), and whole grains (8.3%), no significant associations were observed between these variables and IC (Table 4).

**Table 4:** Prevalence of intestinal constipation and its association with water intake and fiber-rich food sources in adults with obesity class II and III (n = 150)

Variables	Frequency	Prevalence of constipation	PR (CI95%)	*p*-value
	n (%)	n (%)		
Liters of water per day				0.687*
> 2 L	66 (44.00)	15 (22.73)	1.06 (0.46–2.46)	
2 L	28 (18.67)	6 (21.43)	1	
< 2 L	56 (37.33)	16 (28.57)	1.33 (0.58–3.04)	
Raw salad^1^				0.251*
No	81 (54.00)	23 (28.40)	1.40 (0.78–2.51)	
Yes	69 (46.00)	14 (20.29)	1	
Cooked/braised vegetables^1^				0.369*
No	109 (72.67)	29 (26.61)	1.36 (0.68–2.74)	
Yes	41 (27.33)	8 (19.51)	1	
Fresh fruits^1^				0.710*
No	110 (73.33)	28 (25.45)	1.13 (0.58–2.19)	
Yes	40 (26.67)	9 (22.50)	1	
Whole grains^1^				0.057**
No	126 (84.00)	35 (27.78)	3.33 (0.85–13.00)	
Yes	24 (16.00)	2 (8.33)	1	

^*^Pearson's χ^2^ test. **Fisher's exact test. PR: prevalence ratio. 95%CI: 95% confidence interval. ^1^Accessed as daily consumption (yes or no)

After the multiple regression analysis, the following variables were associated with IC: polypharmacy (PR: 2.99, 95% CI: 1.18–7.57, *p* = 0.021), being a former smoker (PR: 3.24, 95% CI: 1.28–9.14, *p* = 0.014) and age (between 18 and 29 years) (PR: 3.12, 95% CI: 1.21–8.06, *p* = 0.019). Non-consumption of whole grains showed a borderline significance (PR: 2.92, 95% CI: 1.00–8.49, *p* = 0.050) (Table 5).

**Table 5:** Multiple regression analysis of the association between intestinal constipation with sociodemographic, lifestyle variables, presence of morbidities and food consumption in adults with obesity class II and III (n = 150)

Variables	Adjusted PR (CI95%)	*p*-value*
Age group		
18–29	3.12 (1.21–8.06)	**0.019**
30–39	1	
40–49	0.82 (0.26–2.57)	0.737
≥ 50	0.95 (0.15–5.85)	0.955
Smoking status		
Nonsmoker	1	
Ex-smoker	3.24 (1.28–9.14)	**0.014**
Smoker	2.30 (0.83–6.38)	0.109
Ingested grams of ethanol (n = 80)		
3–14.99 g	2.19 (0.79–6.06)	0.130
≥ 15 g	1	
Dyslipidemias		
No	1	
Yes	0.49 (0.18–1.33)	0.161
Polypharmacy		
No	1	
Yes	2.99 (1.18–7.57)	**0.021**
Whole grains^1^		
No	2.92 (1.00–8.49)	**0.050**
Yes	1	

Bold: significative result

*Wald statistic. PR: prevalence ratio. 95% CI: 95% confidence interval. ^1^Accessed as daily consumption (yes or no)

## Discussion

In the present study, a high prevalence of constipation was observed in obese classes II and III individuals. Moreover, a worrying intestinal health profile was identified regarding the low weekly frequency of bowel movements, hard stools and the need for excessive effort to evacuate. Constipation was associated with polypharmacy, age and smoking and the non-consumption of whole grains showed a borderline level of significance. The paucity of evidence on constipation in obese or severe obese individuals reinforces the relevance of this study [52, 53].

The prevalence of constipation in our participants with obesity class II and III was high compared to the overall prevalence of constipation in adults of 16% [3] and the prevalence of 5.4% from a population-based cohort study [54]. A population study conducted in the Southern of Brazil found a prevalence closer to the present study i.e. 20.5% in obese individuals including class I obesity (BMI ≥ 30 kg/m^2^) [55]. Other Brazilian population-based studies have shown a prevalence of constipation between 14 and 25% [6–8].

Recent reviews on constipation do not address the prevalence among obese individuals and the factors associated with it in these individuals [3, 30, 31, 56]. There are only few studies on constipation in this specific population [52, 53], which makes it difficult to compare our results. To the best of our knowledge, no previous study has assessed stool consistency using the Bristol Scale in individuals with obesity class II and III. However, a study carried out in Chilean adults found a prevalence of 19.38% of hard stools indicative of intestinal constipation (stools type 1 and 2) [57] while in the present study, this frequency was relatively low (6.67%). A study with morbidly obese patients in the context of bariatric surgery evaluating defecation disorders, which is different from constipation [53], applied the Wexner Constipation Score ≥ 5 and found a constipation prevalence of 20% in 139 patients [53].

We observed in the present study the occurrence of several important characteristics associated to intestinal malfunction, such as: low weekly frequency of bowel movements, hard stools and the need for excessive effort to evacuate. These characteristics culminate in excessive effort when defecating, which can cause several physiological damages such as the weakening of the pelvic floor, excessive perineal descent, rectal intussusception, among others [4]. To investigate intestinal habits is essential to carry out appropriate interventions, since changes in intestinal health culminate in damage to the general state of health and quality of life [4, 56].

In the present study, constipation was associated with age, with the highest prevalence of constipation in the younger age group, contradicting findings in the literature on non-obese individuals that show a higher prevalence in older adults [13]. Studies carried out with non-obese individuals reported decreased bowel movements with increasing age, probably due to low fiber consumption, physical inactivity and hereditary factors [3, 14, 58]. The age group with the highest prevalence of constipation in the severely obese was those aged 50 years or over, similarly to a population study with adult Australian women [59]. This difference in the age range associated with constipation can be attributed to the age range of the present study and also the studied population being comprised of individuals with obesity class II and III.

The association between constipation and smoking is in line with a systematic review on the physiological effects of smoking cessation [60]. Despite all the benefits for the cardiovascular, pulmonary system and reduced risk of cancer, some side effects associated to smoking cessation have been reported, including constipation [60, 61]. There is a lack of specific studies including obese individuals.

The use of medication is associated with several gastrointestinal side effects, constipation being one of the most important, which increases with polypharmacy. Associations between polypharmacy and constipation has been described in previous studies, mainly with older adults, who frequently take several medications [12, 62]. Certain drug groups such as antidepressants, benzodiazepine derivatives, furosemide, levothyroxine sodium and ibuprofen have been associated with constipation [12, 63]. In this study, polypharmacy was associated with constipation. It is known that adults with obesity class II and III often have other associated morbidities, being candidates for the use of several medications and, consequently, side effects such as constipation.

Previous evidence has showed that constipation is more common in women and the probable explanation being the presence of increased hormonal factors during the third and last phase of the menstrual cycle (estrogenic phase) [3, 14, 32, 55, 64]. However, in the present study there was no significant association between constipation and sex, although a higher prevalence was observed in women (26.56%) compared to men (13.64%).

Studies conducted in the general population observed an association of constipation with sociodemographic variables such as having a black or brown skin, low income and low level of education [14, 55]. There was no previous study evaluating the association of constipation with these variables in adults with obesity class II and III. However, our study did not find any significant association between constipation and sociodemographic variables.

The level of obesity class was not associated with constipation in this study, a result that is in line with a systematic review showing that obesity was not associated with constipation [30, 65], as well as a population-based study on various gastrointestinal disorders. All individuals in the present study were obese with a very high BMI value, however, obesity class II, III and severe obesity were not associated with a higher occurrence of constipation.

Our findings did not show a significant association between constipation and the food consumption variables investigated, which is in agreement with a population-based cohort study that found no association between ultra-processed foods and constipation [54]. However, we would like to highlight that, in the present study, the prevalence of constipation was higher in those who did not consume whole grains, fresh fruits and raw salad daily, which are foods rich in fiber, as well as those with inadequate water intake. A diet rich in fiber and adequate water intake contribute to better intestinal functioning, the pH of the colon and the production of by-products with important physiological functions. Therefore, a diet rich in fiber and adequate water intake are important for general health and the dietary treatment of constipation and obesity [66, 67].

We observed a low consumption of fiber-rich foods among the severely obese, reaching 84% of the participants. This finding is in accordance with the literature because fiber intake is inversely associated with body weight and body fat [66, 68]. Obesity and low consumption of fiber-rich foods like vegetables are associated with colon cancer [26], especially in individuals with constipation, causing damage to the intestinal mucosa.

A potential limitation of the present study could be attributed to memory bias due to the use of the Food Frequency Questionnaire (FFQ), since it is an instrument that relies on the individual's memory. The FFQ shows a dietary pattern in a given period and, in this study, the usual food consumption for the last year was evaluated. This, however, is an inherent limitation of food consumption assessment instruments. However, it could have resulted in a lack of association between constipation and food consumption, especially fiber-rich foods. There is still no superior method used in research to assess habitual food consumption. However, to minimize this source of bias and improve data reliability, some precautions were taken before and during data collection, such as training the nutritionist team and standardizing the application of the FFQ. Another potential limitation refers to the sample age range. Our results should be interpreted with caution when extrapolated to older adults because we have included people aged between 18 and 64 years.

The diagnosis and treatment of constipation in obese people are particularly important and can prevent other diseases. Improving both constipation and obesity also means having much lower treatment costs by intervening before the worsening or occurrence of other more serious illnesses. It is important that health professionals assess the presence of constipation in severely obese individuals. However, regardless of the diagnosis, nutritional guidelines such as the intake of more than two liters of water per day and a diet rich in fiber (25–30 g/day) are crucial to prevent the problem [2]. Other measures include, prescription of fiber supplements, magnesium hydroxide and olive oil before starting prescription medications for the treatment of constipation. Regular physical activity is also very effective in improving bowel function [2]. Future research should include lifestyle variables, presence of other morbidities, water and food consumption, in addition to information on the role of the composition of the microbiota in constipation to increase our knowledge on the subject.

In summary, constipation prevalence was high in adults with obesity class II and III. The factors associated with constipation were age, being a former smoker and polypharmacy. The level of obesity, physical activity level, consumption of fiber-rich foods and water intake were not associated with constipation. Considering the fast increase in the prevalence of obesity class II and III the findings from this study could help guiding the treatment in this population.

## Data Availability

The datasets used and/or analyzed during the current study are available from the corresponding author on reasonable request.

## References

[1] Chatoor D, Emmnauel A (2009). Constipation and evacuation disorders. Best Pract Res Clin Gastroenterol.

[2] World Gastroenterology Organisation Global Guidelines. Constipation: a global perspective. 2010

[3] Forootan M, Bagheri N, Darvishi M (2018). Chronic constipation: a review of literature. Medicine (Baltimore).

[4] Bharucha AE, Pemberton JH, Locke GR (2013). American gastroenterological association technical review on constipation. Gastroenterology.

[5] Werth BL, Williams KA, Fisher MJ, Pont LG (2019). Defining constipation to estimate its prevalence in the community: results from a national survey. BMC Gastroenterol.

[6] Chinzon D, Dias-Bastos TRP, da Silva AM, Eisig JN, Latorre MDRDDO (2015). Epidemiology of constipation in São Paulo, Brazil: a population-based study. Curr Med Res Opin..

[7] Schmidt FMQ, de Gouveia Santos VLC, de Cássia DR, Neves JMJ (2016). Constipation: prevalence and associated factors in adults living in Londrina. South Braz Gastroenterol Nurs.

[8] Schmidt FMQ, Santos VLCDG, Domansky RDC, Barros E, Bandeira MA, Tenório MADM (2015). Prevalence of self-reported constipation in adults from the general population. Rev da Esc Enferm da USP.

[9] Rao SSC, Meduri K (2011). What is necessary to diagnose constipation?. Best Pract Res Clin Gastroenterol.

[10] Jiang Y, Tang Y, Lin L (2020). Clinical characteristics of different primary constipation subtypes in a Chinese population. J Clin Gastroenterol.

[11] Staller K, Barshop K, Kuo B, Ananthakrishnan AN (2018). Depression but not symptom severity is associated with work and school absenteeism in refractory chronic constipation. J Clin Gastroenterol.

[12] Fosnes GS, Lydersen S, Farup PG (2011). Constipation and diarrhoea-common adverse drug reactions? A cross sectional study in the general population. BMC Clin Pharmacol..

[13] Gallegos-Orozco JF, Foxx-Orenstein AE, Sterler SM, Stoa JM (2012). Chronic constipation in the elderly. Am J Gastroenterol.

[14] Iraji N, Keshteli AH, Sadeghpour S, Daneshpajouhnejad P, Fazel M, Adibi P (2012). Constipation in Iran: Sepahan systematic review no. 5. Int J Prev Med..

[15] Rao SSC, Rattanakovit K, Patcharatrakul T (2016). Diagnosis and management of chronic constipation in adults. Nat Rev Gastroenterol Hepatol.

[16] Peppas G, Alexiou VG, Mourtzoukou E, Falagas ME (2008). Epidemiology of constipation in Europe and Oceania: a systematic review. BMC Gastroenterol.

[17] World Health Organization (2008). Global strategy on diet, physical activity and health.

[18] Riaz H, Khan MS, Siddiqi TJ, Usman MS, Shah N, Goyal A (2018). Association between obesity and cardiovascular outcomes. JAMA Netw Open.

[19] Barberio AM, Alareeki A, Viner B, Pader J, Vena JE, Arora P (2019). Central body fatness is a stronger predictor of cancer risk than overall body size. Nat Commun.

[20] Di Cesare M, Bentham J, Stevens GA, Zhou B, Danaei G, Lu Y (2016). Trends in adult body-mass index in 200 countries from 1975 to 2014: A pooled analysis of 1698 population-based measurement studies with 19.2 million participants. Lancet.

[21] World Health Organization (2000). Obesity: preventing and managing the global epidemic.

[22] Malta DC, Santos MAS, Andrade SSCDA, Oliveira TP, Stopa SR, Oliveira MMD (2016). Tendência temporal dos indicadores de excesso de peso em adultos nas capitais brasileiras, 2006–2013. Cien Saude Colet.

[23] Lauby-Secretan B, Scoccianti C, Loomis D, Grosse Y, Bianchini F, Straif K (2016). Body fatness and cancer—viewpoint of the IARC working group. N Engl J Med.

[24] Silveira EA, Kliemann N, Noll M, Sarrafzadegan N, Oliveira C (2020). Visceral obesity and incident cancer and cardiovascular disease: An integrative review of the epidemiological evidence. Obes Rev..

[25] Sundbøll J, Thygesen SK, Veres K, Liao D, Zhao J, Gregersen H (2019). Risk of cancer in patients with constipation. Clin Epidemiol.

[26] Alsheridah N, Akhtar S (2018). Diet, obesity and colorectal carcinoma risk: results from a national cancer registry-based middle-eastern study 11 medical and health sciences 1117 public health and health services. BMC Cancer.

[27] Sundbøll J, Thygesen SK, Liao KVD, Zhao J, Gregersen H, Sørensen HT (2019). Risk of cancer in patients with constipation. Clin Epidemiol.

[28] Bouchoucha M, Fysekidis M, Julia C, Airinei G, Catheline JM, Reach G, et al. Functional Gastrointestinal Disorders in Obese Patients. The Importance of the Enrollment Source. Obes Surg. 2015;25: 2143–2152. doi:10.1007/s11695-015-1679-6 10.1007/s11695-015-1679-6 25904236

[29] Delgado-Aros S, Locke GR, Camilleri M, Talley NJ, Fett S, Zinsmeister AR (2004). Obesity is associated with increased risk of gastrointestinal symptoms: a population-based study. Am J Gastroenterol.

[30] Eslick GD (2012). Gastrointestinal symptoms and obesity: a meta-analysis. Obes Rev.

[31] Mugie SM, Benninga MA, Di Lorenzo C (2011). Epidemiology of constipation in children and adults: a systematic review. Best Pract Res Clin Gastroenterol.

[32] Schmidt FMQ, De Gouveia Santos VLC (2014). Prevalence of constipation in the general adult population: an integrative review. J Wound, Ostomy Cont Nurs.

[33] Tantawy SA, Kamel DM, Abdelbasset WK, Elgohary HM (2017). Effects of a proposed physical activity and diet control to manage constipation in middle-aged obese women. Diabetes Metab Syndr Obes Targets Ther.

[34] Aparecida-Silveira E, de Souza JD, dos Santos-Rodrigues AP, Lima RM, de Souza-Cardoso CK, de Oliveira C (2020). Effects of extra virgin olive oil (EVOO) and the traditional Brazilian diet on Sarcopenia in severe obesity: a randomized clinical trial. Nutrients..

[35] Silveira EA, de Souza-Rosa LP, de Carvalho-Santos ASEA, de Souza-Cardoso CK, Noll M (2020). Type 2 diabetes mellitus in class II and III obesity: prevalence, associated factors, and correlation between glycemic parameters and body mass index. Int J Environ Res Public Health..

[36] Rodrigues APS, Rosa LPS, Silva HD, Silveira-Lacerda EDP, Silveira EA. The single nucleotide polymorphism PPARG2 Pro12Ala affects body mass index, fat mass, and blood pressure in severely obese patients. J Obes. 2018;2018. doi:10.1155/2018/2743081 10.1155/2018/2743081 PMC6311828 30652031

[37] Santos ASAC, Rodrigues APS, Rosa LPS, Sarrafzadegan N, Silveira EA (2020). Cardiometabolic risk factors and Framingham risk score in severely obese patients: baseline data from DieTBra trial. Nutr Metab Cardiovasc Dis.

[38] Cardoso CKDS, Santos ASEADC, Rosa LPDS, Mendonça CR, Vitorino PVDO, Peixoto MDRG (2020). Effect of extra virgin olive oil and traditional brazilian diet on the bone health parameters of severely obese adults: a randomized controlled trial. Nutrients.

[39] Santos ASEADC, Rodrigues APDS, Rosa LPDS, Noll M, Silveira EA (2020). Traditional Brazilian diet and olive oil reduce cardiometabolic risk factors in severely obese individuals: a randomized trial. Nutrients.

[40] Sturm R (2007). Increases in morbid obesity in the USA: 2000–2005. Public Health.

[41] WHO. The WHO STEPwise approach to chronic disease risk factor surveillance. WHO STEPS Surveill Man. 2005; 490.

[42] Lima MCP, Kerr-Côrrea F, Rehm J (2013). Alcohol consumption pattern and coronary heart disease risk in Metropolitan São Paulo: analyses of GENACIS project. Rev Bras Epidemiol.

[43] Masnoon N, Shakib S, Kalisch-Ellett L, Caughey GE (2017). What is polypharmacy? a systematic review of definitions. BMC Geriatr.

[44] Francisco PMSB, Azevedo-Barros MBD, Segri NJ, Alves MCGP, Cesar CLG, Malta DC (2011). Comparação de estimativas para o auto-relato de condições crônicas entre inquérito domiciliar e telefônico - Campinas (SP). Brasil. Rev Bras Epidemiol..

[45] Harrison C, Britt H, Miller G, Henderson J (2014). Examining different measures of multimorbidity, using a large prospective cross-sectional study in Australian general practice. BMJ Open.

[46] Zigmond AS, Snaith RP (1983). The hospital anxiety and depression scale. Acta Psychiatr Scand.

[47] Furlan-Viebig R, Pastor-Valero M (2004). Development of a food frequency questionnaire to study diet and non-communicable diseases in adult population. Rev Saude Publica.

[48] Koloski NA, Jones M, Young M, Talley NJ (2015). Differentiation of functional constipation and constipation predominant irritable bowel syndrome based on Rome III criteria: a population-based study. Aliment Pharmacol Ther.

[49] Longstreth GF, Thompson WG, Chey WD, Houghton LA, Mearin F, Spiller RC (2006). Functional bowel disorders. Gastroenterology.

[50] Yurtdaş G, Acar-Tek N, Akbulut G, Cemali Ö, Arslan N, Beyaz Coşkun A (2020). Risk factors for constipation in adults: a cross-sectional study. J Am Coll Nutr.

[51] Lewis SJ, Heaton KW (1997). Stool form scale as a useful guide to intestinal transit time. Scand J Gastroenterol.

[52] Poylin V, Serrot FJ, Madoff RD, Ikrumuddin S, Mellgren A, Lowry AC (2011). Obesity and bariatric surgery: a systematic review of associations with defecatory dysfunction. Color Dis.

[53] Sileri P, Franceschilli L, Cadeddu F, De Luca E, D’Ugo S, Tognoni V (2012). Prevalence of defaecatory disorders in morbidly obese patients before and after bariatric surgery. J Gastrointest Surg.

[54] Schnabel L, Buscail C, Sabate JM, Bouchoucha M, Kesse-Guyot E, Allès B (2018). Association between ultra-processed food consumption and functional gastrointestinal disorders: results from the French NutriNet-Santé Cohort. Am J Gastroenterol.

[55] Collete VL, Araújo CL, Madruga SW (2010). Prevalência e fatores associados à constipação intestinal: Um estudo de base populacional em Pelotas, Rio Grande do Sul, Brasil, 2007. Cad Saude Publica.

[56] Bharucha AE, Wald A (2019). Chronic constipation. Mayo Clin Proc.

[57] Godoy ZJ, Morales OMDLÁ, Schlack VC, Papuzinski AC (2011). Prevalencia de constipación y su asociación con enfermedades crónicas en Centro de Salud Familiar Marcos Maldonado, Viña del Mar, 2010. Rev ANACEM.

[58] Kaboli SA, Pourhoseingholi MA, Moghimi-dehkordi B, Safaee A (2010). Factors associated with functional constipation in Iranian adults: a population-based study. Gastroenterol Hepatol from bed to bench.

[59] Chiarelli P, Brown W, McElduff P (2000). Constipation in Australian women: prevalence and associated factors. Int Urogynecol J.

[60] Gratziou C (2009). Respiratory, cardiovascular and other physiological consequences of smoking cessation. Curr Med Res Opin.

[61] Çan G, Öztuna F, Topbaş M (2007). Complaints related to smoking cessation. Tuberk Toraks.

[62] Komiya H, Umegaki H, Asai A, Kanda S, Maeda K, Nomura H (2019). Prevalence and risk factors of constipation and pollakisuria among older home-care patients. Geriatr Gerontol Int.

[63] Fosnes GS, Lydersen S, Farup PG (2012). Drugs and constipation in elderly in nursing homes: what is the relation?. Gastroenterol Res Pract..

[64] Garrigues V, Gálvez C, Ortiz V, Ponce M, Nos P, Ponce J (2004). Prevalence of constipation: agreement among several criteria and evaluation of the diagnostic accuracy of qualifying symptoms and self-reported definition in a population-based survey in Spain. Am J Epidemiol.

[65] Le Pluart D, Sabaté JM, Bouchoucha M, Hercberg S, Benamouzig R, Julia C (2015). Functional gastrointestinal disorders in 35 447 adults and their association with body mass index. Aliment Pharmacol Ther.

[66] Slavin JL (2005). Dietary fiber and body weight. Nutrition.

[67] Stewart ML, Nikhanj SD, Timm DA, Thomas W, Slavin JL (2010). Evaluation of the effect of four fibers on laxation, gastrointestinal tolerance and serum markers in healthy humans. Ann Nutr Metab.

[68] Davis JN, Alexander KE, Ventura EE, Toledo-Corral CM, Goran MI (2009). Inverse relation between dietary fiber intake and visceral adiposity in overweight Latino youth. Am J Clin Nutr.

